# ﻿*Pyruszhaoxuanii* (Rosaceae), A new pear species from Danxiashan Mountain, Guangdong, China

**DOI:** 10.3897/phytokeys.254.138039

**Published:** 2025-03-28

**Authors:** Xiao-Wei Yi, Ying-Yu Wu, Qiang Fan, Fang Chen, Zai-Xiong Chen, Bin-Bin Liu, Cui-Ying Huang

**Affiliations:** 1 State Key Laboratory of Biocontrol and Guangdong Provincial Key Laboratory of Plant Stress Biology, School of Life Sciences, Sun Yat-sen University, Guangzhou 510275, China; 2 National Park and Nature Education Research Institute, Sun Yat-sen University, Guangzhou 510275, China; 3 Guangdong Danxiashan National Nature Reserve Administration, Shaoguan 512300, China; 4 State Key Laboratory of Plant Diversity and Specialty Crops, Institute of Botany, Chinese Academy of Sciences, Beijing 100093, China; 5 China National Botanical Garden, Beijing 100093, China

**Keywords:** Danxia landscape, new species, phylogeny, *Pyrus*

## Abstract

*Pyruszhaoxuanii* is described as a new species from Guangdong Province, China, within the genus *Pyrus*, specifically under P.subg.Pashia. Although it shares morphological similarities with *P.calleryana*, *P.zhaoxuanii* can be distinguished by its uniquely small, obovate, leathery leaves, which have an obtuse apex and short petioles. A phylogenetic analysis based on single nucleotide polymorphisms (SNPs) indicated that *P.zhaoxuanii* forms an independent branch within *Pyrus* and is categorized in the Oriental clade, P.subg.Pashia. Currently, this species has only been recorded in the Danxiashan National Nature Reserve. Considering its potential distribution and population size, we recommend classifying this species as Least Concern (LC) according to the IUCN Red List classifications and criteria.

## ﻿Introduction

The genus *Pyrus* L. belongs to the apple tribe Maleae in the Rosaceae family (Zhang et al. 2017; Liu et al. 2020a, 2022; Wang et al. 2024). Currently, at least 25 confirmed species of *Pyrus* have been identified worldwide, with 14 species recorded in China (Gu and Spongberg 2003; Jin et al. 2024a). As one of the most important fruit trees, pears have a cultivation history spanning possibly 3,000 years (Pu and Wang 1963; Teng 2011). *Pyrus* species exhibit self-incompatibility, necessitating cross-pollination from different flowers, which increases genetic heterozygosity among individual plants. Interspecific hybridization is common within *Pyrus*, leading to a blend of genes and extensive genetic variation among species (Jin et al. 2024b). This frequent introgression is prevalent in the apple tribe Maleae, including apples, pears, and their relatives (Phipps et al. 1991; Lo and Donoghue 2012; Liu et al. 2019, 2020b, 2023; Jin et al. 2023). This genetic complexity complicates determining relationships between different *Pyrus* species (Westwood and Bjornstad 1971), making their classification particularly challenging. Furthermore, many geographical subspecies have historically been regarded as “species” (Westwood and Challice 1978).

*Pyruscalleryana* Decne., commonly known as Callery pear, is native to eastern and southern China, Korea, and Japan (Bell and Zimmerman 1990; Gu and Spongberg 2003) and is frequently used as a rootstock for pear trees. The Flora of China recognizes four varieties of *P.calleryana*: P.calleryanavar.calleryana, P.calleryanavar.integrifolia T.T.Yu, P.calleryanavar.koehnei (C.K.Schneid.) T.T.Yu, and P.calleryanavar.lanceolata Rehder (Gu and Spongberg 2003).

To clarify the classification of the genus *Pyrus*, a previous study examined the diversity of *Pyrus* and the independent domestication of Asian and European pears (Wu et al. 2018). This study analyzed 113 individuals and constructed a phylogenetic tree of major *Pyrus* species, categorizing them into Asian and European pears, resulting in six distinct groups. Among these, *P.calleryana*, *P.pashia* Buch.-Ham. ex D.Don, and *P.betulifolia* Bunge were classified as Asian pear Group II, indicating their close phylogenetic relationships. These three species exhibit relatively limited domestication and share characteristics of small, undomesticated fruits. Building on this research, an updated infrageneric classification divided pears into two subgenera: P.subg.Pashia (Asian pears) and P.subg.Pyrus (European pears) (Jin et al. 2024a). All members of Group II fall under P.subg.Pashia.

During our investigation in Danxiashan Mountain, we found two types of Callery pear (*P.calleryana*) with different morphological characteristics. Individuals on the gentle slopes at the foot of the mountain are consistent with the normal *P.calleryana* phenotype morphologically, while those on the steep slopes and cliffs exhibit distinct traits, including small, obovate, thick, leathery leaves with obtuse tips and short petioles. Additionally, some branchlets have evolved into thorns, and the plant’s stature has become shrubbier.

Initially, we hypothesized that these plants were ecotypes of *P.calleryana.* However, as our research progressed, it became clear that this plant should be classified under P.subg.Pashia as a new branch in the phylogenetic tree. Based on morphological characteristics and phylogenetic analyses, we propose that it represents a new species, which we describe and illustrate here.

## ﻿Methods and materials

### ﻿Samples collection, DNA extraction, and sequencing

**Figure 1.**
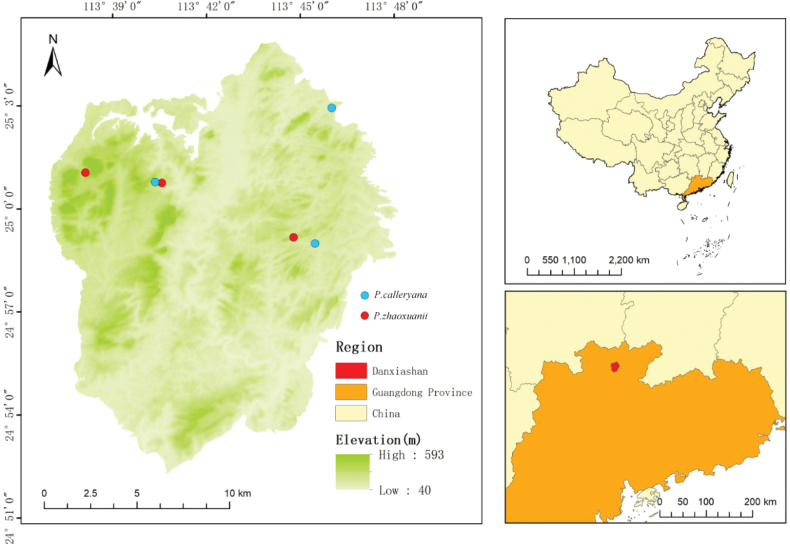
Distribution of *Pyruszhaoxuanii* and *Pyruscalleryana* in Danxiashan Mountain.

Four individuals of the putative new species and six individuals of *Pyruscalleryana* were collected from four locations in Mount Danxiashan, Renhua County, Shaoguan City, Guangdong Province (Table 1, Fig. 1). Fresh leaf material from each individual was dried and stored in silica gel. Total genomic DNAs were extracted using CTAB method (Doyle and Doyle. 1987). The extracted genomic DNAs were assessed for integrity, purity, and concentration using agarose gels and Qubit 4.0 with Qubit^®^ DNA Assay Kit (Life Technologies). The qualified DNA samples (≥ 50 ng) were then sent to Jierui Biotech (Guangzhou, China) for paired-end library preparation, followed by genome skimming sequencing on Illumina Xplus (Illumina Inc.; San Diego, California, USA), adhering to the standard Illumina sequencing protocol. Voucher specimens for each individual were deposited in the herbarium of Sun Yat-sen University (SYS).

**Table 1.** Sample collection information.

Pop.ID	Collection number	Location	Geographical ordination	individual
L1	101	Shaoshishan	24°58'15"N, 113°44'50"E	2
S1	102	Shaoshishan	24°58'29"N, 113°44'10"E	2
L2	201	Bazhai	25°00'27"N, 113°39'54"E	2
S2	202	Bazhai	25°00'24"N, 113°40'07"E	1
L3	301	Heshangzhai	25°02'09"N, 113°45'45"E	1
L4	401	Yanyan	25°00'54"N, 113°37'42"E	1
S4	402	Yanyan	25°00'54"N, 113°37'42"E	1

*The Pop.ID “L” means “large leaves” representing *P.calleryana*; “S” means “small leaves” representing *P.zhaoxuanii*.

### ﻿Morphological study

The morphological study documented images of the new species during its flowering and fruiting stages. The morphological characteristics of the putative new species were compared with those of *P.calleryana*, as well as with specimens from other species within the *Pyrus* genus. The plant specimens used in this study were obtained from the herbaria P, PE, SYS, NAS that herbarium acronym as per BIEN 4.2 (https://bien.nceas.ucsb.edu/bien/data-contributors/herbaria/). Voucher specimens are preserved in the herbarium of Sun Yat-sen University (SYS).

### ﻿Phylogenetic analyses

#### ﻿Other nuclear genome data acquisition

We downloaded re-sequencing data of the nuclear genome of 22 individuals, including 20 representative species of *Pyrus*, such as *P.pashia* and *P.betulifolia*, as well as the closely related genus, *Maluspumila*, from the NCBI nucleotide database (Suppl. material 1). The fasterq-dump program in SRA Toolkit v. 3.1.0 (https://github.com/ncbi/sra-tools/wiki/Home) was used to convert the SRA-formatted files to paired-end FASTQ format files.

#### ﻿Constructing a phylogenetic tree based on nuclear genome data

The raw data from the ten individuals sequenced in our study, together with the genomic data for 22 other species downloaded from NCBI, were used to reconstruct a phylogenetic tree. The genome of *Pyrusbretschneideri* Rehder (GeneBank sequence number: GCF_019419815.1) served as the reference. The FASTQ formatted paired-end sequencing data (1 and 2) were mapped to the reference genome using BWA v. 0.1.17 (Li 2013). The resulting mapping files were converted into BAM format using SAMtools v. 1.6 (Danecek et al. 2021), and the mapping results were sorted while removing PCR duplicate sequences. BCFtools v. 1.9 (Danecek et al. 2021) was employed to generate a set of candidate variant positions (SNPs and indels), followed by variant detection. The VCF file was normalized and filtered with the parameters “-s LOWQUAL -e ‘QUAL<20 || INFO/DP <5’ ” to remove variants with quality values (QUAL) less than 20 or depth (DP) less than 5. All single nucleotide polymorphisms (SNPs) in the VCF file were extracted, and the SNP data of the 32 individuals were combined into one file and filtered twice. The parameters were set to “-i ‘DP>=5 & DP<=100 & QUAL>=30’ -s LOWQUAL” and “-i ‘MAF>=0.05’” to retain sites with a DP between 5 to 100, QUAL of at least 30, and a minor allele frequency of at least 5%. A custom Perl script was utilized to select sites present in at least 75% of the individuals. The VCF file was converted to PHYLIP format using vcf2phylip.py (Ortiz 2019), and the phylogenetic tree was reconstructed using IQ-TREE v. 2.1.4 (Minh et al. 2020) based on the maximum likelihood method. The parameters were set to “-m MFP+ASN -bb 2000” (Kalyaanamoorthy et al. 2017), and the best fitting model for DNA replacement was determined to be TVM+I+R4, calculated using the Bayesian information criterion.

## ﻿Results and discussion

**Figure 2.**
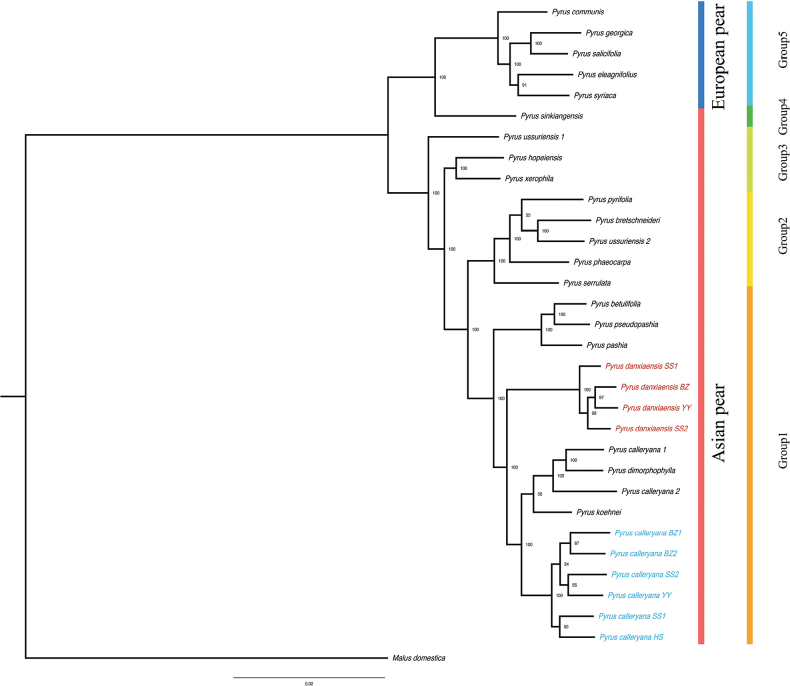
Phylogenetic tree built based on SNPs, *Maluspumila* as the outgroup. Among them, those marked red are *P.zhaoxuanii*, and those marked blue are *P.calleryana* that we collected in Danxia Mountain. The numbers displayed on the diagram represent the bootstrap confidence level of the phylogenetic tree.

A total of 1,164,026 SNPs were generated for the 32 samples. The phylogenetic tree inferred from these SNPs closely resembled findings from previous SNP-based research (Wu et al. 2018), defining five subclades with high support values (Fig. 2). Furthermore, our results also correspond with the ortholog-based phylogenomic studies (Jin et al. 2024a, 2024b). Those *P.calleryana* individuals we collected, along with its varieties, formed a sister group to the putative new species. Ultimately, the putative new species, *P.calleryana*, *P.pashia*, *P.betulifolia*, and *P.pseudopashia*, collectively formed Group 1, which showed high support values and belonged to the P.subg.Pashia (Jin et al. 2024a). This phylogenetic tree indicates that pronounced genetic differentiation exists between the new species and other species within Group 1.

The putative new species is most similar to the *Pyruscalleryana*, both morphologically and molecularly. Both species possess small fruits, five white petals, and corymb inflorescences. However, compared to *P.calleryana*, the putative new species has smaller leaves (19–31 × 10–15 mm vs. 40–80 × 35–60 mm), obovate leaves (vs. broadly ovate or ovate, rarely narrowly elliptic), apex obtuse (vs. apex acuminate, rarely acute), shorter petioles (2–12 mm vs. 20–40 mm), distinct stem thorns (vs. nearly no stem thorns), and a shrubby habit (vs. tree habit) (Table 2).

**Table 2.** Morphological comparison of *P.zhaoxuanii*, and *P.calleryana*.

Feature	*P.zhaoxuanii*	*P.calleryana*
Leaf Size	19–31 × 10–15 mm	40–80 × 35–60 mm
Leaf Texture	Leathery, thick	Papery, thin
Leaf Shape	obovate, rarely elliptical	Elliptical or ovate
Leaf Apex	blunt, rarely acuminate	Acuminate, rarely acute
Leaf Base	Cuneate	Round to wide cuneate
Inflorescence Number	2–7(8)	6–12
Pedicel	Tomentose	Glabrous
Petiole	Short	Long
Branch Thorn	0–11	0–4
Mean Branch Thorn	5	0.2

*The Pop.ID “L” means “large leaves” representing *P.calleryana*; “S” means “small leaves” representing *P.zhaoxuanii*.

Morphologically, a common characteristic of Group 1 in our study is small fruit size, which distinguishes them from other groups. Within Group 1, *Pyruszhaoxuanii* differentiates from other species due to its small, obovate leaves. When we first encountered it at Mount Danxia, we suspected it was an ecotype of *P.calleryana*. However, *P.zhaoxuanii* has established a new branch on the phylogenetic tree based on SNP data. Molecular phylogenetics supports its classification as an independent species rather than an ecotype of *P.calleryana*.

### ﻿Taxonomic treatment

#### ﻿ Pyrus zhaoxuanii

Taxon classificationPlantaeRosalesRosaceae

X.W.Yi, B.B.Liu & Q.Fan sp. nov.

6062E3BB-7F95-5180-8689-E38F7AEE8B2E

urn:lsid:ipni.org:names:77359363-1

**Figure 3.**
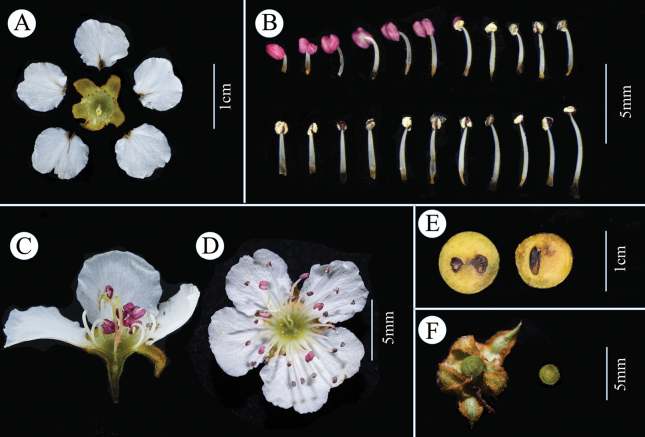
Flower and pome of *Pyruszhaoxuanii***A** frontal dissection of the flower **B** stamens **C** longitudinal section of the flower **D** front view of the entire flower **E** cross and longitudinal sections of the fruit **F** cross-section of the ovary. Scale bars: 1 cm (**A, E**); 5 mm (**B, C, D, F**).

**Figure 4.**
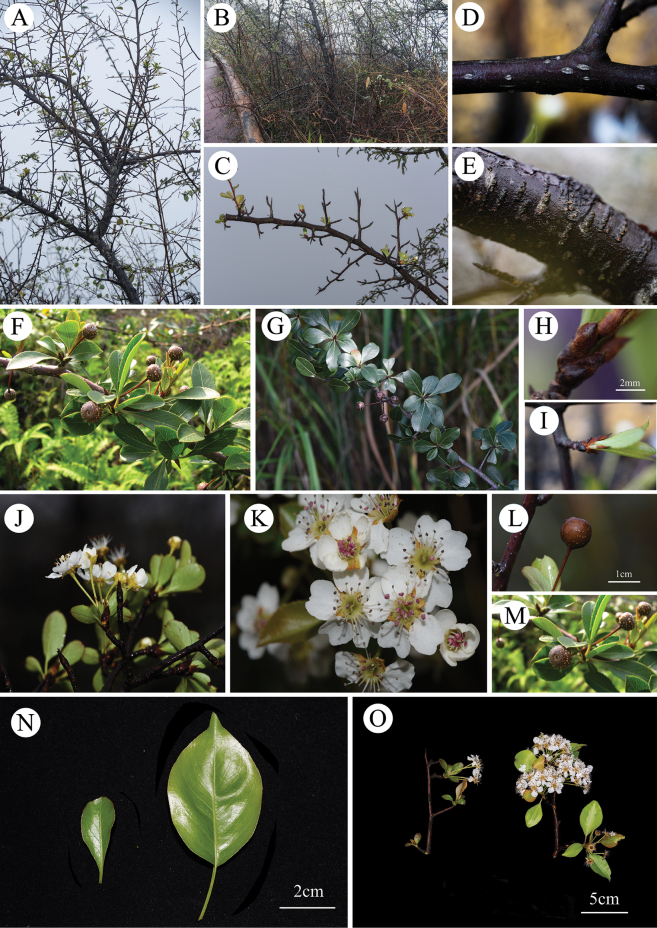
*Pyruszhaoxuanii***A** plant, stem with many branches and thorn **B** habitat, growing on the edge of cliffs or steep slopes **C** branches with many subdivisions and thorns **D** lenticels on young branches **E** surface of the stem **F, G** leaves during the fruiting period **H, I** leaf buds and bud scales with hair **J, K** flowers **L, M** fruits **I** comparison of *P.zhaoxuanii* and *P.calleryana* (left: *P.zhaoxuanii*, right: *P.calleryana*).

**Figure 5.**
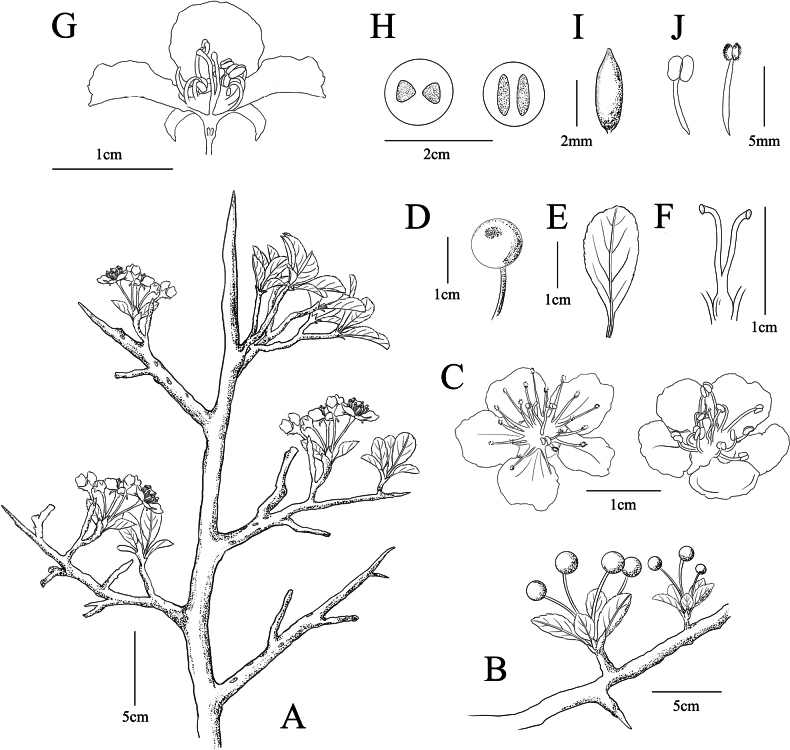
Line drawing of *Pyruszhaoxuanii***A** flowering plant **B** fruiting plant **C** flower **D** appearance of fruit **E** leaf **F** pistil **G** longitudinal section of flower **H** longitudinal and cross section of fruit **I** bud scales **J** stamens. Scale bars: 5 cm (**A, B**); 1 cm (**C, D, E, F, G**); 2 cm (**H**); 2 mm (**I**); 5 mm (**J**). Illustrated by Si-Rui Pan.

**Figure 6.**
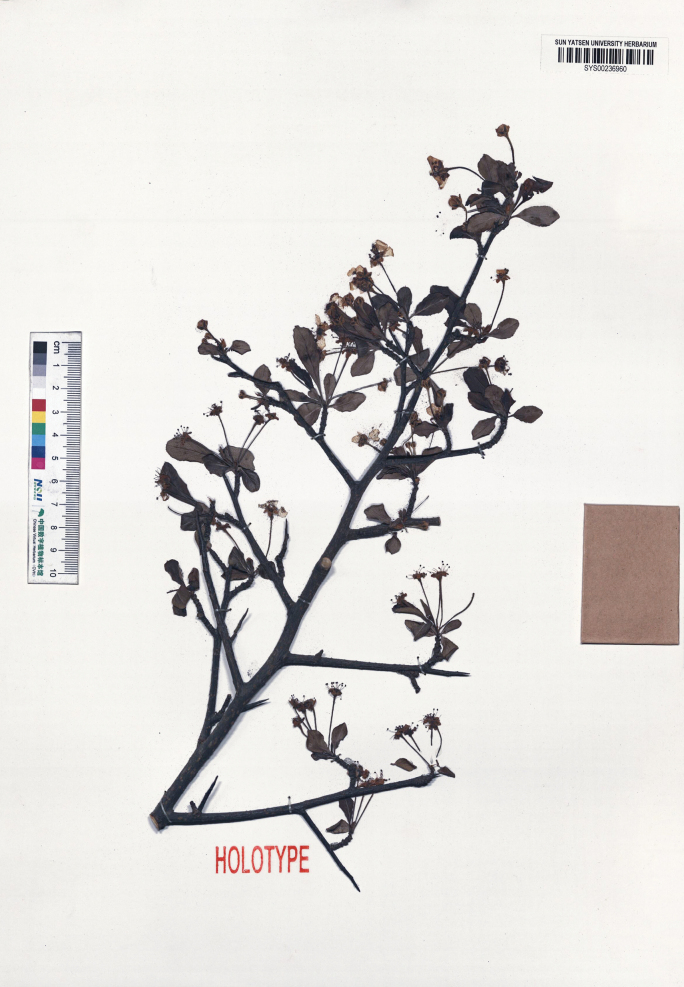
Holotype of *Pyruszhaoxuanii*, *Y.Y. Wu et al. DNPC4016* (SYS).

Figs 3 , 4 , 5 , 6 Chinese name. 昭璇梨

##### Type.

China. • Guangdong Province, Shaoguan City, Danxiashan National Nature Reserve, 25°0'28.26"N, 113°39'42.80"E, alt. 380 m, 24 February 2024, *Y.Y. Wu et al. DNPC4016* (holotype: SYS!; isotypes: SYS!, PE!).

##### Diagnose.

*Pyruszhaoxuanii* is similar to *P.calleryana*, but can be differentiated by its small, obovate leaves, short petioles, pronounced stem thorns, and shrubby habit.

##### Description.

Deciduous shrubs or small trees, 2–5 m high, with lateral branches; bark dark gray to brownish with vertical splits; much-branched; Twigs smooth, spiny, covered with linear lenticels. Leaf buds long ellipsoid, with 5–7 hairy scales outside. Leaves fascicled on short branches; petiole 2–12 mm long; Leaf blade obovate, rarely elliptic, (14) 19–31 (45) × (7) 10–15 (23) mm, margin obtusely serrate, base cuneate, apex blunt or round, rarely acuminate, leathery in quality. Corymb 2–8 flowered, receptacle cup-shaped, covered with short hairs; sepals are triangular, ca. 4 mm long, woolly, with rust-colored velutinous on the margins, bending downwards at the apex; flower 12–17.5 (29.5) mm in diameter; Petals 5, 5–8 × 5–6 mm, pure white, glabrous, obovate, margin slightly sinuate, apex rounded; filament 3–7 mm long; anthers purple to pink; style 2, 4–6.5 mm long, glabrous; flower stalk (19) 21–35 mm long. Pome small, spheroid, reddish-brown to brownish-black, ca. 1 cm long; fruiting pedicel 18–36 mm long. Seeds ovate, blackish, ca. 2 × 5 mm.

##### Phenology.

Flowering was observed from February to March, while fruiting occurred from September to October.

##### Etymology.

*Pyruszhaoxuanii* is named in honor of Prof. Zhao-Xuan Zeng (1921–2007), a famous geographer of South China Normal University, who made significant contribution to the study of danxia landscape.

##### Distribution and habitat.

The new species is currently known only from its type locality, Mount Danxiashan, Renhua County, Guangdong Province, China. It typically grows on steep slopes at altitudes of 200–600 m above sea level.

##### Conservation status.

The new species is a common shrub found on the steep slopes of Mount Danxia. Most individuals are located within the Danxia Nature Reserve, which is well protected, and we observed no active threats or ongoing declines in population size. According to the Guidelines for Using the IUCN Red List Categories and Criteria, v. 16 (IUCN Standards and Petitions Committee 2024), we suggest classifying *P.zhaoxuanii* as Least Concern(LC).

##### Additional specimens examined

**(Paratypes)**: China. • Guangdong Province, Shaoguan City, Danxiashan National Nature Reserve, 113°39'50.56"N, 25°0'27.81"E, alt. 328 m, 24 February 2024, *Y.Y. Wu et al. DNPC4014* (SYS!); China. • Guangdong Province, Shaoguan City, Danxiashan National Nature Reserve, 24°58'28.73"N, 113°44'10.29"E, alt. 285 m, 26 September 2023, *Y.Y. Wu & Q. Fan 102* (SYS!).

## Supplementary Material

XML Treatment for Pyrus zhaoxuanii

## ﻿Additional information

### Author ORCIDs

Xiao-Wei Yi https://orcid.org/0009-0004-8867-7418

Qiang Fan https://orcid.org/0000-0003-4254-6936

Bin-Bin Liu https://orcid.org/0000-0002-0297-7531

Cui-Ying Huang https://orcid.org/0009-0009-6134-2032

### Data availability

All of the data that support the findings of this study are available in the main text or Supplementary Information.

## Supplementary materials

Supplementary material 1 GeneBank accession numbers of the sampled species used in this study Data type docx https://binary.pensoft.net/file/1294176This dataset is made available under the Open Database License (http://opendatacommons.org/licenses/odbl/1.0/). The Open Database License (ODbL) is a license agreement intended to allow users to freely share, modify, and use this Dataset while maintaining this same freedom for others, provided that the original source and author(s) are credited.Xiao-Wei Yi, Ying-Yu Wu, Qiang Fan, Fang Chen, Zai-Xiong Chen, Bin-Bin Liu, Cui-Ying Huang
