# Supplementary material for: ﻿Pyruszhaoxuanii (Rosaceae), A new pear species from Danxiashan Mountain, Guangdong, China
Source: PhytoKeys. 2025 Mar 28;254:161–73. doi: 10.3897/phytokeys.254.138039 (PMC11971636; doi:10.3897/phytokeys.254.138039)
Supplement: Supplementary material 1 — GeneBank accession numbers of the sampled species used in this study [file phytokeys-254-161_article-138039__-s001.docx]

| ScientificName | SRA accession |
| --- | --- |
| *P._betulifolia* | SRR25212413 |
| *P._serrulata* | SRR25212412 |
| *P._ussuriensis* | SRR25212408 |
| *P._communis* | SRR16954066 |
| *P._pashia* | SRR22103107 |
| *P._phaeocarpa* | SRR22103105 |
| *P._ussuriensis* | SRR22103103 |
| *P._elaegnifolius* | SRR22103102 |
| *P._georgica* | SRR22103100 |
| *P._salicifolia* | SRR22103110 |
| *P._syriaca* | SRR22103109 |
| *P._pyrifolia* | SRR23268750 |
| *P._bretschneideri* | SRR27896858 |
| *P. hopeiensis* | SRR14318819 |
| *P. koehnei* | SRR7135513 |
| *P. xerophila* | SRR7135605 |
| *P. pseudopashia* | SRR7135523 |
| *P. sinkiangensis* | SRR7135563 |
| *P._dimorphophylla* | SRR7135500 |
| *Malus_pumila* | SRR23461136 |
| *P._calleryana1* | SRR16505594 |
| *P._calleryana2* | SRR7135498 |

**Appendix 1.** GeneBank accession numbers of the sampled species used in this study
